# Development of a real time PCR assay using SYBR Green chemistry for bovine leukemia virus detection

**DOI:** 10.1186/1742-4690-8-S1-A17

**Published:** 2011-06-06

**Authors:** Gonzalo Rama, Gonzalo Moratorio, Gonzalo Greif, Gonzalo Obal, Sergio Bianchi, Lorena Tomé, Federico Carrion, Ana Meikle, Otto Pritsch

**Affiliations:** 1 Laboratorio de Técnicas Nucleares, Facultad de Veterinaria, Universidad de la República, Lasplaces, Montevideo, 1550, Uruguay; 2 Unidad de Biofísica de Proteínas, Institut Pasteur de Montevideo, Montevideo, 11400, Uruguay; 3 Laboratorio de Virología Molecular. Centro de Investigaciones Nucleares. Facultad de Ciencias, Universidad de la República, Montevideo, 11400, Uruguay; 4 Unidad de Biología Molecular, Institut Pasteur de Montevideo, Montevideo, 11400, Uruguay; 5 Departamento de Fisiopatología, Hospital de Clínicas, Facultad de Medicina, Universidad de la República, Montevideo, 11600, Uruguay; 6 Departamento de Inmunobiología, Facultad de Medicina, Universidad de la República, Montevideo, 11800, Uruguay

## 

In Uruguay, more than 50% of dairy cattle individuals are infected by Bovine Leukemia Virus (BLV). A main goal of our country is to decrease this extremely high prevalence by developing efficient eradication programs for this disease.

The aim of this study was to develop a rapid and sensitive real time PCR assay using SYBR green chemistry to detect and quantify BLV proviral DNA by amplifying gp51 gene from bovine peripheral blood.

By using plasmid containing gp51 gene diluted in non-infected bovine genomic DNA we could determine the assay sensitivity. A comparative analysis with validated diagnostic tests (AGID, ELISA and direct nested PCR) was performed in 45 dairy cattle samples. All AGID positive animals (n=14) were positive by ELISA, while three negative AGID samples were also positive by ELISA. All ELISA positive animals (n=17) were positive by nested PCR. Real-time PCR technique shown that 15 out of 17 positive ELISA samples were positive whereas 10 out of 28 negative ELISA samples were also positive. These results reveal high agreement with nested PCR, and confirm an increased sensitivity of the PCR (real-time and nested) over the ELISA and AGID tests respectively.

Overall, our results show that this SYBR Green -based PCR assay may be a useful, simple, and rapid tool to detect BLV infection in dairy cattle samples that could be adapted to high-troughput diagnostic procedures.

